# Incidence of heterotopic ossification following hip arthroscopy is low: considerations for routine prophylaxis

**DOI:** 10.1007/s00264-022-05402-4

**Published:** 2022-04-28

**Authors:** Zaki Arshad, Henry David Maughan, Malgorzata Garner, Erden Ali, Vikas Khanduja

**Affiliations:** 1 School of Clinical Medicine, University of Cambridge, Cambridge Biomedical Campus, Box 111, Cambridge, CB2 0SP UK; 2 Young Adult Hip Service, Department of Trauma & Orthopaedics, Addenbrooke’s - Cambridge University NHS Foundation Trust, Hills Road, Cambridge, CB2 0QQ UK; 3 Department of Surgery, Division of Trauma & Orthopaedics, University of Cambridge, The Old Schools Lane, Cambridge, UK

**Keywords:** Hip arthroscopy, Heterotopic ossification, Scoping review, Prophylaxis

## Abstract

**Purpose:**

This scoping review aims to map and summarise the available literature on heterotopic ossification (HO) following hip arthroscopy, with particular focus on incidence, distribution as per Brooker classification, efficacy of prophylactic measures and factors that may influence the likelihood of production of HO.

**Methods:**

A computer-based search was performed on PubMed, Embase, Emcare, Cinahl, ISI web of science and Scopus using the terms ‘heterotopic ossification’ and ‘hip arthroscopy’. Articles reporting heterotopic ossification following hip arthroscopy for any condition were included after two-stage title/abstract and full-text screening.

**Results:**

Of the 663 articles retrieved, 45 studies were included. The proportion of patients with HO ranged from 0 to 44%. The majority of the cases were either Brooker grade I or II. Of the six studies investigating the effect of NSAID prophylaxis, five reported a significantly lower incidence of heterotopic ossification associated with its use. Weak evidence suggests that an outside-in arthroscopic approach, no capsular closure, male sex and mixed cam and pincer resection may be associated with an increased risk of HO.

**Conclusion:**

Although there is a large variation in rates of HO following hip arthroscopy in the current literature, the majority of studies report a low incidence. Evidence exists advocating the administration of post-operative NSAIDs to reduce the incidence of HO following hip arthroscopy. This, combined with the low risk of complications, means there is a favourable risk–benefit ratio for prophylactic NSAID used in HA. Future research should work to identify patient clinical and demographic factors which may increase the risk of development of HO, allowing clinicians to risk stratify and select only specific patients who would benefit from receiving NSAID prophylaxis.

**Supplementary Information:**

The online version contains supplementary material available at 10.1007/s00264-022-05402-4.

## Introduction

Hip arthroscopy (HA) has become increasingly popular over the last two decades [1, 2]. It is now used successfully in the diagnosis and treatment of a variety of soft tissue and osseous and intra- and extra-articular hip conditions such as femoroacetabular impingement syndrome (FAIS), subspinous impingement, ischiofemoral impingement, developmental dysplasia of the hip, iliopsoas impingement, deep gluteal syndrome, external snapping hip syndrome and trochanteric bursitis [3–15]. It is a relatively safe procedure with a relatively low risk of complications. Complications associated with traction-/pressure-related injuries, iatrogenic chondral and labral injury, fluid extravasation and instrument breakage are discussed in detail in the literature [16–19]. However, there is evidence highlighting the increased incidence of heterotopic ossification (HO) following HA and its impact. There are studies reporting rates as high as 44% in the literature [20, 21].

HO is presumed to be caused following soft tissue injury and a subsequent inflammatory cascade. The resultant inflammatory environment leads to angiogenesis, progenitor cell differentiation and ectopic bone formation from differentiated cell types [22, 23]. Whilst Brooker grades I and II HO may be asymptomatic, grades III and IV may present with stiffness, pain and reduced range of motion [24, 25]. Some evidence exists highlighting the role of non-steroidal anti-inflammatory drugs (NSAIDs) in the reduction of HO following hip arthroscopy [22, 24, 26]. This effect is thought to occur through modulation of osteoprogenitor cells and interference of cell signalling pathways [27, 28]. However, the debate on routine prophylaxis for HO following HA continues.

This scoping review aims to map and summarise the available literature on HO following hip arthroscopy, with particular focus on incidence, distribution as per Brooker classification, efficacy of prophylactic measures and factors that may influence the likelihood of production of HO. By doing so, we aim to enhance the reader’s understanding of this common complication, inform clinical decision-making with regard to the use of prophylactic measures and critically identify areas for future research. We hypothesise that whilst the incidence of HO may be increasing, the overall incidence remains low and that prophylaxis may not be required for all patients.

## Materials and methods

The methodological framework for scoping reviews was first outlined by Arksey and O’Malley in 2005 and more recently updated by Levac et al. and The Joanna Briggs Institute [29–31]. These articles outline five common steps in a scoping review, all of which are outlined below.

### Identifying the research question

The following review questions were developed:

1. What is the incidence of HO following hip arthroscopy?
2. What prophylactic measures are reported and what is their efficacy?
3. In which locations does HO occur?
4. What is the distribution of severity in terms of the Brooker classification?
5. What patient- and treatment-related factors are associated with the development of HO?

### Identification of relevant studies

Free text and medical subject heading (MeSH) terms including ‘heterotopic ossification’, ‘hip arthroscopy’ and ‘femoroacetabular impingement’ were used, with the Boolean operators ‘and’, ‘or’ used to combine search terms as appropriate. The full search strategy is available in the Appendix. The search was initially performed in PubMed, before being adapted for and used in five other databases including, OVID Embase, OVID Emcare, Scopus, ISI Web of Science and CINAHL. All searches were computer-based and performed on 17th January 2021. Reference list checking was also performed using review articles identified by the above search process.

### Study selection

Selected manuscripts were imported into Rayyan systematic reviews web application (Qatar Computing Research Institute, Doha, Qatar) for screening and selection [32]. A two-stage title/abstract and full-text screening was performed by two authors independently, using the outlined selection criteria:

- Participants: Patients undergoing hip arthroscopy for any condition.
- Intervention*:* Any form of hip arthroscopy.
- Control: No specific control or comparison group was required for inclusion in this review.
- Outcome: The primary outcome of interest was incidence of HO. Other outcomes of interest included: Brooker classification, location of HO, effect of prophylactic treatments and factors associated with the development of HO.
- Study design: Original research observational studies, cohort studies and randomised control trials were included. Review articles, case reports, commentaries, letters to the editor and abstracts were excluded.
- Date: No specific date restrictions were imposed
- Language: English language

Studies describing treatment of heterotopic ossification were also included as were those that reported no complications following hip arthroscopy, with the assumption that no patients showed signs of HO. Studies which reported results of any hip arthroscopy procedure yet did not specifically describe the formation of HO were excluded. Studies reporting a mini-open approach or the use of both an arthroscopic and open treatment were also excluded. Non-English articles were excluded at the full-text screening stage rather than through imposing limits on the database search. This allowed the display of these potentially relevant foreign language articles in an appendix, to ensure transparency.

Differences in opinion regarding the inclusion or exclusion of articles were first resolved by discussion between the two authors and, failing this, by consultation with a third author.

### Charting the data

A data extraction form was created in Microsoft Excel, with the following column headings used to extract data from all included studies:

- Author
- Year
- Type of study
- Number of hips and number of patients
- Patients mean age
- Patient-sex ratio
- Indication for arthroscopy
- Prophylactic measures used
- Incidence of HO
- Brooker classification
- Location of HO
- Effect of any prophylactic measures used
- Treatment of HO
- Factors associated with the development of HO
- Follow-up mean and range

### Collating, summarising and reporting results

**Fig. 1:**
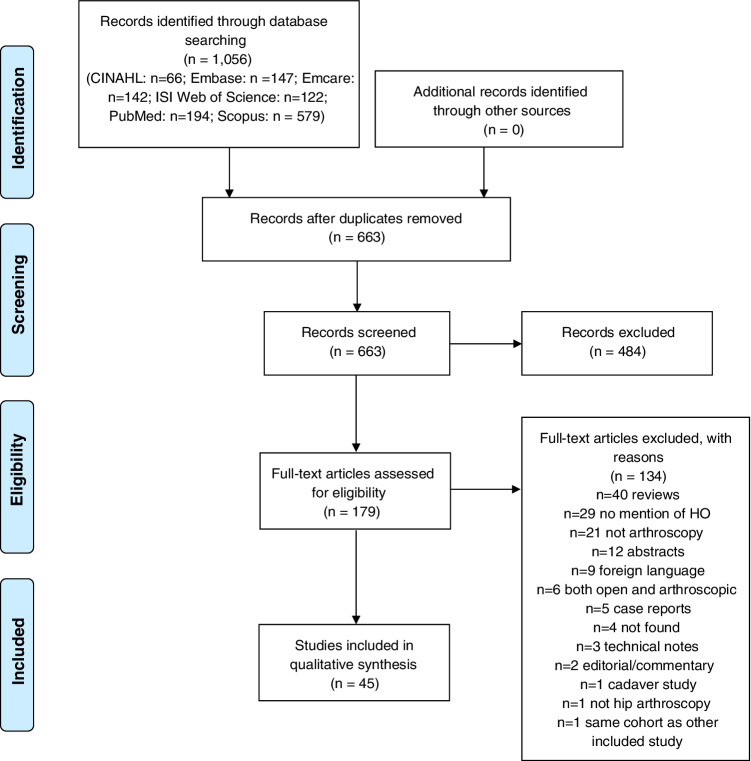
PRISMA flow diagram displaying the number of studies retrieved and removed at each screening stage

**Fig. 2:**
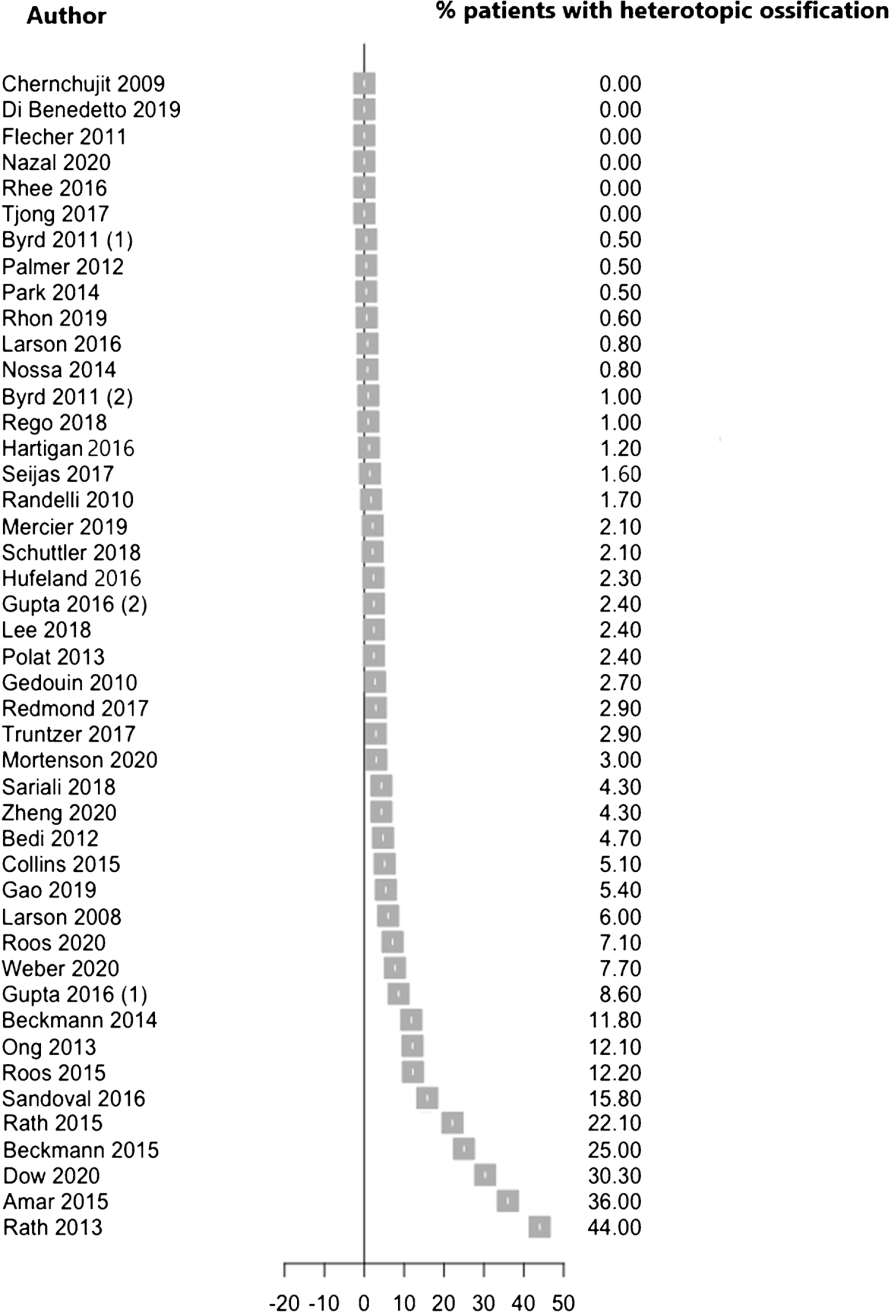
Forest plot showing the overall percentage of patients in each study who developed heterotopic ossification following hip arthroscopy. This allows the visualisation, in one figure, of heterotopic ossification rates reported in all included studies

The number of studies retrieved and removed following each screening stage are shown in the PRISMSA flow diagram (Fig. 1) [33]. Study characteristics, including first author, type of study, number of hips and patients, patient age and sex and follow-up period are shown in Table 1. Forest plots generated using R studio are used to display the incidence of HO in included studies (Fig. 2). The distribution of severity of HO according to the Brooker classification is displayed in Table 2. A qualitative thematic approach was used throughout, with results reported according to the key themes described, including incidence of HO, Brooker classification, prophylactic measure used and their effects and factors associated with the development of HO. This approach is commonly used in scoping reviews and aids in the identification and mapping of key themes within a broad topic [31, 34].

**Table 1:** Summary of the first authors, year of publication, type of study, number of hips, mean patient age, patient-sex ratio and follow-up period reported by all included studies. *Pts* patients, *M* male, *F* female, *RCT* randomised controlled trial, *NA* not available. *: In these cases the number of males to females refers to the number of hips rather that the number of patients

Author	Year	Type of study	Number of hips	Mean age in years (range)	Sex ratio M/F	Mean follow-up in months (range)
Amar [35]	2015	Cohort	100	37.5 (18–68)	61:39	12.7 (6–23)
Beckmann [36]	2014	Cohort	288	31.4	116:172	Minimum 6
Beckmann [26]	2015	RCT	106	35	39:67	10.7
Bedi [24]	2012	Cohort	616	31.3	342:274	24
Byrd (1) [37]	2011	Case series	200	28.6 (11–60)	148:52	19 (12–60)
Byrd (2) [38]	2011	Case series	100	34 (13–76)	67:33	24
Chernchujit [39]	2009	Case series	7	23 ± 12	5:2	15.7
Collins [40]	2015	Cohort	39	39.6 (22–64)	16:23	31.2 (24–67.2)
Di Benedetto [41]	2019	Case series	13	65 (47–82)	9:4	10 (3–12)
Dow [42]	2020	Cohort	454	39	226:228	6 months (all) 12 months (419) 24 months (304)
Flecher [43]	2011	Case series	23	34 (17–54)	14:9	21 (12–28)
Gao [44]	2019	Case series	242	26.2 ± 9.5	140:102	23 (11–34)
Gedouin [45]	2010	Case series	111 (110 pts)	31 (16–49)	78:32	10 (6–14)
Gupta (1) [46]	2016	Case series	70	36.4 (16.8–70.2)	31:39	28 (20–47.4)
Gupta (2) [47]	2016	Case series	595	38 (13.2–76.4)	228:367	29 (24–66)
Hartigan [48]	2016	Case series	82 (78 pts)	23 (14.9–39.8)	25:57*	39 (22–77.6)
Hufeland [49]	2016	Case series	44	34.3 (17–65)	24:20	66.5 ± 14.5
Larson [50]	2008	Case series	100 (96 pts)	34.7	54:42	9.9 (3–36)
Larson [51]	2016	Case series	1615	30.5 912–76)	810:905	18.7 (6–53)
Lee [52]	2018	Case series	41	34.6 916–54)	21:20	92.4 (85–117)
Mercier [53]	2019	Cohort	47 (43 pts)	33 (15–65)	32:11	30.6 (14–58)
Mortensen [54]	2020	Cohort	233	33.1	85:148	13.4 ± 9.4
Nazal [55]	2020	Case series	14	32.7 (16–55)	6:8	80
Nossa [56]	2014	Case series	362 (360 pts)	40.4 (15–79)	147:215	Minimum 6
Ong [57]	2013	Case series	66	38 (15–68)	30:36	28 (24–36)
Palmer [58]	2012	Case series	201 (185 pts)	40.2 (14–87)	99:102 *	
Park [59]	2014	Case series	200 (197 pts)	44.6 (19–70)	97:100	28.2 (19–42)
Polat [60]	2013	Case series	42	35.1 (16–52)	25:17	28.2 (10–72)
Randelli [61]	2010	Cohort	300	37.4 (16–66)	180:120	17.9 (6–36)
Rath [21]	2013	Case series	50	36.7	31:19	29.6 (9–62) weeks
Rath [62]	2015	Cohort	163	36.6 918–68)	91:72	12.9 (4–23)
Redmond [63]	2017	Case series	23	38.6	10:13	18
Rego [64]	2018	Case series	198 (102 receiving arthroscopy)	33 (18–49)	112:86	59 (24–132)
Rhee [65]	2016	RCT	37 (30 pts)	34.3	15:22 *	32.1 (25.5–41.2)
Rhon [66]	2019	Case series	1870	32.2	1038:832	24
Roos [67]	2015	Case series	41 (40 pts)	36.1 (21–47)	36:4	29.1 (12–36)
Roos [68]	2020	Case series	28 (25 pts)	32.1 (19–44)	18:7	29.5 (6–82)
Sandoval [69]	2016	Cohort	101 (91 pts)	37 (15.7–59.6)	58:33	22 (12–40)
Sariali [70]	2018	Case series	47	36 ± 12	NA	39.6 ± 12
Schuttler [71]	2018	Case series	529	43.9	254:275	Minimum 6 weeks
Seijas [72]	2017	Case series	258	36.6	137:121	Minimum 12
Tjong [73]	2017	Case series	106 (86 pts)	38.1 (17–59)	36:50	37.2 (28–79)
Truntzer [74]	2017	Case series	2581	NA	968:1613	12
Weber [75]	2020	Case series	39	19.5	29:10	23.5
Zheng [76]	2020	Case series	327	36.3 (14–69)	226:101	39.4 (24–80)

**Table 2:** Showing a breakdown of the Brooker classification of heterotopic ossification cases in included studies

Author	Brooker Grade				
-	I	II	III	IV	Total
Amar [35]	17 (47.2%)	15 (32.6%)	4 (11.1%)	0 (0%)	36
Beckmann [36]	23 (67.6%)	9 (26.5%)	2 (5.9%)	0 (0%)	34
Beckmann [26]	17 (70.8%)	7 (29.2%)	0 (0%)	0 (0%)	24
Bedi [24]	18 (62.1%)	4 (13.8%)	6 (20.7%)	1 (3.4%)	29
Dow [42]	68 (73.9%)	20 (21.7%)	4 (4.3%)	0 (0%)	92
Gao [44]	9 (69.2%)	3 (23.1%)	1 (7.7%)	0 (0%)	13
Gedouin [45]	0 (0%)	2 (66.7%)	1 (33.3%)	0 (0%)	3
Hufeland [49]	0 (0%)	1 (100%)	0 (0%)	0 (0%)	1
Larson [51]	13 (100%)	0 (0%)	0 (0%)	0 (0%)	13
Mortensen [54]	7 (100%)	0(0%)	0 (0%)	0 (0%)	7
Ong [57]	6 (75%)	1 (12.5%)	1 (12.5%0	0 (0%)	8
Palmer [58]	0 (0%)	0 (0%)	1 (100%)	0 (0%)	1
Rath [21]	13 (59.1%)	5 (22.7%)	4 (18.2%)	0 (0%)	22
Rath [62]	17 (47.2%)	15 (32.6%)	4 (11.1%)	0 (0%)	36
Redmond [63]	23 (100%)	0 (0%)	0 (0%)	0 (0%)	23
Roos [67]	4 (80%)	0 (0%)	1 (20%)	0 (0%)	5
Roos [68]	2 (100%)	0 (0%)	0 (0%)	0 (0%)	2
Zheng [76]	10 (71.4%)	4 (28.6%)	0 (0%)	0 (0%)	14
**Total**	**247 (68.0%)**	**86 (23.7%)**	**29 (8.0%)**	**1 (0.3%)**	**363**

### Quality assessment and risk of bias

Although the assessment of quality of the studies and risk of bias forms a key part of systematic reviews, these steps are not required in a scoping review [29–31].

## Results

The search strategy outlined, resulted in the identification of 663 unique articles, of which 45 (6.8%) were finally included.

Most articles (33/45, 73.3%) were level IV evidence, whilst ten level III studies (22.2%) and two level I randomised control trials (4.4%) were also included (Table 1). The total number of hips was 12,613 (12,538 patients) and the mean pooled age was 34.4 years. A forest plot displaying the overall incidence of HO is shown (Fig. 2). Rates of HO vary widely from 0 to 44%. Of the included studies, 14 (31.1%) report an incidence of HO of 1% or less, whilst a total of 30 (66.7%) studies report an incidence of under 5% and 36 (80%) studies less than 10%. Unfortunately, it was not possible to pool individual study results to derive an overall incidence due to low level of evidence included and inherent risk of bias and heterogeneity associated with such study designs [77, 78].

Of the included studies, 19 reported a severity stratification of HO cases in terms of the Brooker classification (Table 2). All included studies, except for Gedouin et al. and Hufeland et al. report the majority of HO cases were Brooker Grade 1, whilst only one study describes a case of Brooker grade 4 HO [24, 49, 79]. It was not considered appropriate to pool data on the distribution of Brooker grade across studies due to the low level of evidence included.

### Prophylactic measures

A total of 16 included studies (34.8%) reported on the prophylactic use of NSAIDs (naproxen, celecoxib, indomethacin or aspirin). Six studies directly compared the development of HO in those receiving NSAID prophylaxis to controls receiving no prophylaxis. A summary of these studies is shown in Table 3. Of these, five reported a significantly reduced incidence of HO in those receiving NSAIDs compared to those receiving no treatment. Two studies, Bedi et al. (2012) and Randelli et al. (2010), compare the efficacy of two different NSAID regimens [24, 61].

**Table 3:** Results of those studies comparing heterotopic ossification occurrence in patients given prophylaxis to those receiving no prophylaxis or comparing heterotopic ossification development after the use of two different NSAID regimes. *PO* by mouth, *BD* twice a day, *QD* once daily, *RR* risk ratio

Author	Group 1 (number of hips)	Group 2 (number of hips)	Ho_1_	HO_2_	Effect of prophylaxis
Beckmann [26]	Naproxen 500 mg PO BD, 3 weeks (48)	Placebo (48)	2/48 (4.2%)	22/48 (45.8%)	RR 0.09 for HO in group 1 compared to group 2 (*P* < 0.001)
Beckmann [36]	Naproxen 500 mg PO BD, 3 weeks (196)	No prophylaxis (92)	11/196 (5.6%)	23/92 (25.0%)	Ho 13.6 times more likely in no prophylaxis group (*P* = 0.003)
Bedi [24]	Naproxen 500 mg PO BD, 30 days (277)	Indomethacin 75 mg QD, 4 days, followed by naproxen 500 mg PO BD for 30 days and omeprazole 20 mg daily for first 4 days (339)	23/277 (8.3%)	6/339 (1.8%)	Ho 4.6 times more likely in group 1 (*P* < 0.05). no significant difference between the groups in the likelihood of developing HO with a Brooker grade > 1
Dow [42]	Celecoxib 400 mg QD, 6 weeks (243)	No prophylaxis (211)	30/131 (22.9%) (112 pts lost to follow-up)	62/ 173 (35.8%) (38 pts lost to follow-up)	Significantly reduced incidence of HO in group 1 (*P* < 0.001)
Mortensen [54]	Naproxen 500 mg PO BD, 2 weeks (185)	Naproxen 500 mg PO BD, 3 weeks (48)	5/185 (2.7%)	2/48 (4.2%)	No significant difference in HO incidence between groups
Nossa [56]	Celecoxib 200 mg QD, 3 weeks (122)	No prophylaxis (240)	0%	3/240 (1.3%)	No significant association between prophylaxis sand HO incidence
Randelli [61]	Etoricoxib 90 mg daily, 3 weeks (15), Naproxen 500 mg PO BD, 3 weeks (248), Others—aceclofenac,indomethacin,ketoprofen, 3 weeks (22)	No prophylaxis (15)	0%	5/15 (33.3%)	Significantly higher incidence of HO in Group 2 (*P*,0.001)
Rath [62]	Etodolac 600 mg QD, 2 weeks	No prophylaxis (100)	0%	36/100 (36%)	Significantly lower incidence of HO in group 1 (*P* < 0.001)

### Arthroscopic approach

Three studies directly compared the incidence of HO following different arthroscopic approaches/techniques. Amar et al. (2015) found no significant difference in the incidence of HO between patients receiving capsular closure (14/50, 28%) and those not receiving any capsular closure (22/50,44%) *p* = 0.144 [35]. Similarly, Rhee et al. (2016) found a 0% incidence of heterotopic ossification production in patients where knot tying and knotless suture anchor techniques were utilised [65]. Sandoval et al.(2016) report a significantly higher (*p* = 0.017) incidence of HO with the use of an ‘outside in’ arthroscopic approach (12/53, 22.6%) in comparison to a standard arthroscopic approach (4/48, 8.3%) [69].

### Factors influencing the development of heterotopic ossification

A total of six studies described other factors which may influence the development of HO. A description of these factors is provided in Table 4.

**Table 4:** Summary of factors which may affect the incidence of heterotopic ossification following hip arthroscopy

Author	Factors
Beckmann [36]	In those patients receiving femoral osteoplasty, the degree of resection was significantly higher in those who went on to develop HO (18.9°) compared to those who did not (12.3°), *P* = 0.036. Of those given prophylactic NSAID therapy, 3 patients who underwent capsular repair developed HO, whilst 8 who did not undergo capsular repair developed HO, although this difference was not significant. No significant difference in HO development was found between the first and second 46 cases of the performing surgeon. In a multivariate logistic regression model, mixed type FAI resection was associated with an increased risk of HO compared to CAM only resection (Odds ratio 52.5, *P* < 0.011). No significant associated observed with regards to age or sex
Bedi [24]	Of the 29 cases of HO, 7 occurred in patients undergoing cam femoral osteoplasty, 2 in those receiving isolated acetabular resection for pincer impingement and 20 in those receiving mixed resection. However, no significant association was found between type of procedure and development of HO, likely due to the small numbers involved. Most cases of HO occurred in male patients receiving osteoplasty for FAI, during which the capsule was cut. Multivariate logistic regression found no association between type of procedure and HO development
Dow [42]	OF the 92 cases of HO, significantly more (*P* < 0.001) occurred in males (69/92, 75%) compared to females. This significant difference was also seen specifically in both the treatment and control groups where 75.8% and 73.3% of patients who developed HO were male
Randelli [61]	Significantly lower HO incidence was seen in the NSAID prophylaxis group. However, no significant difference in age, sex, weight or type of procedure performed (pincer rim trimming or cam head neck junction osteoplasty) seen between the treatment group receiving NSAID prophylaxis and controls
Rath [62]	Significantly lower HO incidence was seen in those receiving prophylaxis compared to controls. The latter group also had a significantly longer mean surgery time of 121.9 min, compared to 106.2 min in the control group
Rath [21]	Bivariate logistic backward stepwise regression analysis showed no significant association between sex, diagnosis, procedure performed, anchor use and surgery time and HO development

### Location of heterotopic ossification

Three studies, describing 77 cases of HO also report the location of its development [24, 36, 76]. Beckmann et al. (2014) report all 34 cases of Ho developed anterior to the hip, whilst Bedi et al. (2012) describe 14 anterior and 15 lateral cases, and Zheng et al. (2020) find 13 central cases and one posterior case. Of these cases, 54 (70.1%) occurred anterior to the hip joint, 15 (19.5%) laterally, eight cases (10.4%) anterolaterally and one (1.3%) posteriorly to the hip.

### Treatment of heterotopic ossification

Revision arthroscopy for excision of HO was required in 9/34 (26.5%) of patients who developed HO in the series of Beckmann et al. (2014) [36]. Revision surgery was also reported by other authors: 7/29 (24.1%) of patients in Bedi et al. (2012), 4/14 (28.6%) in Gupta et al. (2016) (2), 3/8 (37.5%) in Ong et al.(2013), 9/92 (9.8%) in Dow et al.(2020) and 2/14 (14.3%) in Zheng et al.(2020) [24, 36, 42, 47, 57, 76].

Revision excision of HO shows encouraging results, with Redmond et al.(2017) reporting statistically significant post-operative increases in terms of mHHS, HOS-ADL, HOS-SS and NAHS scores, along with a statistically significant reduction in VAS values [63]. Similarly, Zheng et al.(2020) report a post-operative increase when assessing mHHS and HOS-ADL values [76].

## Discussion

Our study found that the overall incidence of HO after arthroscopy of the hip is low. Although individual studies report a HO incidence between 0 and 44%, two thirds of included studies describe an incidence of 5% or under and one third report an incidence under 1%. Although no formal meta-analysis was possible, majority of cases were Brooker grade 1 or 2 cases, with more severe cases rarely found in 11 studies. Given the rise in popularity of hip arthroscopy and corresponding increase in publications in its field, it is not surprising that the majority of included articles were published in the last decade [80].

Although there is a large amount of literature concerning the topic of HO following hip arthroscopy, with 45 articles included in this review, almost 75% are level IV evidence, with limited higher levels of evidence as exemplified by the availability of only two randomised control studies. Due to the high degree of heterogeneity present in level III and IV studies, pooling of rates of HO in these studies was not possible, and hence a statistical comparison against the 30 studies not reporting any use of prophylactic measures was not performed.

A large variation in reported rates of HO is seen. The majority (36/45, 80%) of studies report rates of less than 10%, with six articles reporting no cases of HO in their cohorts. However, rates as high as 36% and 44% are reported in the studies of Amar et al.(2015) and Rath et al.(2013), respectively [21, 35]. One reason for this large variation may be the use of prophylactic NSAID therapy to prevent HO. Such a strategy has been described in 16 (34.8%) of the included studies. Another explanation for this variation may be because radiographs are not obtained routinely, six months or a year post-HA in all included studies, which may produce an underestimate of HO incidence.

Of the six studies directly comparing the occurrence of HO in those receiving prophylactic NSAIDs with controls, five report significantly decreased rates in the former group [26, 36, 42, 56, 61, 62]. There is therefore robust evidence suggesting that post-operative prophylactic NSAID administration is effective in reducing the incidence of HO following HA. Nevertheless, questions remain regarding its routine use in all patients undergoing HA, and it is therefore important to carefully consider the risk–benefit ratio. Firstly, the use of NSAID prophylaxis does not completely eliminate the risk of HO, with rates as high as 22.9% and 8.3% seen in the Dow et al.(2020) and Bedi et al.(2012) despite post-operative NSAID administration [24, 42]. Furthermore, the results of this review suggest that the large majority of HO cases may be classified as Brooker Grade I or II, which typically does not present with symptoms and is instead detected on radiographic imaging [24]. There is also currently no evidence to suggest that prophylactic NSAID therapy may decrease the severity of HO according to the Brooker classification, with Beckmann et al.(2015) describing no significant difference in Brooker classification between patients receiving prophylactic NSAIDs and controls receiving no prophylaxis [36]. Furthermore, there is no evidence to suggest that the development of HO affects patient outcomes following hip arthroscopy. Dow et al.(2020) report no significant difference in iHOT33 values between patients with and without HO, whilst no significant difference was also found between the prophylaxis and control group in terms of the proportion of patients achieving MCID at 6 months, 1 year or 2 years post-operatively [42]. Similarly, Zheng et al. (2020) found significant post-operative improvements in mHHHS VAS, HOS-ADL and HOS-SS in those with asymptomatic HO [76].

Whilst NSAID use is cost effective and generally considered safe, complications may occur in some, particularly those with pre-existing cardiovascular or renal risk [81–87]. However, the majority of studies included in this review did not routinely collect or report on side effects or complications associations with NSAID use. Of the two studies that reported this information, one reported a case of acute renal failure, one haematochezia following acute colitis and three cases of gastritis [36], whilst the other reported relatively minor side effects such as headache, weight gain and gastrointestinal upset [26]. It is likely that the patients undergoing hip arthroscopy are in general a healthy population with few concomitant cardiovascular or renal diseases. This combined with the relatively short period of NSAID administration (Table 3) and the lack of evidence suggesting a high risk of serious side effects indicates that the safety profile of NSAIDs should not be a major concern in this clinical context.

Despite our results suggesting the majority of HO are Brooker grade I or II, this does not provide an adequate rationale against routine NSAID prophylaxis as it does not necessarily reflect the proportion of patients requiring revision excision. For example, 7/29 patients in the cohort of Bedi et al.(2012) required revision excision of HO, all of which were either grade I or II cases [24]. Furthermore, re-operation for excision rates of between 9.8% and 37.5% are reported in the included studies. It is thus clear that even a small area of HO, with a Brooker grade of I or II may cause significant symptoms requiring excision [24]. This effect may be explained through consideration of the location of ectopic ossification. For example, small areas of ossification located in the joint capsule, iliopsoas or rectus femoris may cause impingement on movement and hence require excision due to functional deficit and/or pain [24].

One way in the need to prevent HO may be balanced against the potential complications and may be through alterations in dosage. For example, Mortensen et al.(2020) found no significant difference in the rate of HO between those receiving two weeks and three weeks of Naproxen 500 mg PO BD [54]. Careful patient stratification and selection using risk factors for the development of HO may provide another suitable option.

Some studies have suggested factors such as male sex, arthroscopic approach, lack of capsular closure, length of operation and type of procedure (cam resection, pincer resection or mixed resection) may influence the development of HO [24, 35, 36, 42, 62, 69]. However, these factors are derived from small-scale individual studies with low-quality study designs and therefore cannot be used to guide clinical decision-making. This is a key area of further research, with more high-quality comparative studies required in determining patient or treatment-related risk factors for the development of HO. This will not only enable the stratification and selection of appropriate patients for NSAID prophylaxis but also the development of an evidence-based protocol for the prevention of HO development following HA. However, until the influence of these factors can be determined, it is more appropriate to administer prophylactic NSAIDs to all patients undergoing HA, owing to their efficacy at preventing HO and the subsequent need for surgical excision and favourable safety profile. To aid the development of a HO reduction protocol, further research should also investigate other prophylactic techniques, such as radiation therapy. Research suggests this technique is effective in reducing the risk of HO following open total hip arthroplasty; however its use in HA has not been investigated [88, 89].

## Limitations

It is important to be aware of potential differences in radiographic follow-up between studies, which may also partly account for the large variation in rates of HO. Some studies included in this review focus specifically on the incidence of HO after hip arthroscopy, providing patients with regular radiographic imaging, whilst others have a more general aim of reporting outcomes/complications after hip arthroscopy. Our results suggest that the majority of cases of HO are likely to be Brooker grade I or II that is often asymptomatic and detected only through radiographic imaging. It could therefore be the case that the latter group of studies, which often have long periods of clinical follow-up, but might not provide patients with such thorough radiographic follow-up, may provide an underestimate of HO rates. Dow et al. (2020) describe 67 cases of HO after six months radiographic follow-up, 85 after one year and 92 after two years [42]. Although the majority of cases occur in the first six months, an increase is seen across a two year period, despite the loss to follow up of 150 patients. This highlights the effect a potential lack of radiographic follow-up may have on the underestimation of HO rates.

## Conclusion

Although a large variation in rates of HO following HA is seen in the current literature, the majority of studies report a low incidence and most cases described are typically asymptomatic Brooker Grade I or II presentations. Whilst evidence suggests that post-operative NSAIDs do reduce the incidence of heterotopic ossification following hip arthroscopy, their routine administration in all patients undergoing HA needs to be considered in terms of the risk–benefit ratio, specifically given our findings suggesting a low incidence of symptomatic HO. Identification of patient- and treatment-related risk factors associated with the production of heterotopic ossification may allow clinicians to risk stratify patients, thereby aiding in patient selection and decision-making with respect to the use of NSAIDs for prophylaxis. However, as such a strategy cannot yet be implemented in a safe manner, prophylactic NSAIDs should be administered to all patients undergoing HA.

## Supplementary Information

Below is the link to the electronic supplementary material.

Supplementary file1 (DOCX 15 KB)

Supplementary file2 (DOCX 20 KB)

## Data Availability

Not applicable.
