# Supplementary material for: Incidence of heterotopic ossification following hip arthroscopy is low: considerations for routine prophylaxis
Source: Int Orthop. 2022 Apr 28;46(7):1489–500. doi: 10.1007/s00264-022-05402-4 (PMC9166824; doi:10.1007/s00264-022-05402-4)
Supplement: Supplementary file 1 — Supplementary file1 (DOCX 15 KB) [file 264_2022_5402_MOESM1_ESM.docx]

| Search Term | Number of Results |
| --- | --- |
| 1. Hip Arthroscopy AND heterotopic ossification | 126 |
| 1. Hip Arthroscopy AND ossification | 156 |
| 1. Hip Arthroscopy [MesH} AND heterotopic ossification | 108 |
| 1. Heterotopic ossification [MesH] AND hip arthroscopy | 110 |
| 1. (femoroacetabular impingement OR FAI) AND heterotopic ossification | 125 |
| 1. 1 OR 2 OR 3 OR 4 OR 5 | 194 |

Appendix table: Full search strategy used, number of results shown refers to the number of articles retrieved when searching PubMed,
