# Supplementary material for: Incidence of heterotopic ossification following hip arthroscopy is low: considerations for routine prophylaxis
Source: Int Orthop. 2022 Apr 28;46(7):1489–500. doi: 10.1007/s00264-022-05402-4 (PMC9166824; doi:10.1007/s00264-022-05402-4)
Supplement: Supplementary file 2 — Supplementary file2 (DOCX 20 KB) [file 264_2022_5402_MOESM2_ESM.docx]

**Studies Published in a Non-English Language**

[1] Deng X, Liu J, Yang S, Wang X, Li Z. [Application of arthroscopic surgery combined with direct anterior approach in hip diseases]. Zhongguo xiu fu chong jian wai ke za zhi = Zhongguo xiufu chongjian waike zazhi = Chinese J reparative Reconstr Surg 2018;32:1167–71. https://doi.org/10.7507/1002-1892.201803115.

[2] Rühmann O, Wünsch M, Lipka W, Stark DA, Lerch S. [Arthroscopic arthrolysis of the hip]. Oper Orthop Traumatol 2014;26:341–52. https://doi.org/10.1007/s00064-013-0285-9.

[3] Möckel G, Miehlke W. Arthroscopic treatment of psoas impingement . Oper Orthop Traumatol 2018;30:72–9. https://doi.org/10.1007/s00064-018-0535-y.

[4] Xie Z, Jin D, Sheng J, Zhang C. Mid-term effectiveness of surgical hip dislocation for femoroacetabular impingement. Zhongguo Xiu Fu Chong Jian Wai Ke Za Zhi 2019;33:451–4. https://doi.org/10.7507/1002-1892.201811083.

[5] Arthroscopic decompression of extra-articular subspinal hip impingement. Oper Orthop Traumatol 2018:1–11. https://doi.org/http://dx.doi.org/10.1007/s00064-018-0538-8.

[6] Dienst M, Kusma M, Steimer O, Holzhoffer P, Kohn D. [Arthroscopic resection of the cam deformity of femoroacetabular impingement]. Oper Orthop Traumatol 2010;22:29–43. https://doi.org/10.1007/s00064-010-3003-5.

[7] Wünsch M, Rühmann O, Lipka W, Stark DA, Lerch S. [Arthroscopic synovectomy of the hip joint]. Oper Orthop Traumatol 2014;26:469–86. https://doi.org/10.1007/s00064-014-0327-y.

[8] Knoblich A. Hip Arthroscopy Ossification Prophylaxis. Z Orthop Unfall 2016;154:117.

[9] Albillos X, Cuellae AD, Martinez J. Incidence of heterotopic ossification after hip arthroscopy with outside-inside technique. Rev Esp Artrosc y Cir Articul 2018;25:42–7. https://doi.org/http://dx.doi.org/10.24129/j.reaca.25161.fs1709037.

**Studies not found**

[1[ Zsolt Z, András G, Miklós P. Hip arthroscopy: Short-term outcomes . Orv Hetil 2020;161:340–6. https://doi.org/10.1556/650.2020.31668.

[2] Owens BD, Busconi BD. Arthroscopy for hip dislocation and fracture-dislocation. Am J Orthop (Belle Mead NJ) 2006;35:584–7.

[3] Philippon MJ, Schrodere Souza BG, Briggs KK. Hip arthroscopy and labral treatment in patients with femoroacetabular impingement. Minerva Ortop e Traumatol 2009;60:293–302.

[4] Yukizawa Y, Matsuda DK, Sakai A, Uchida S. Hip Arthroscopy for Diffuse Idiopathic Skeletal Hyperostosis Using a Capsulotomy-First Approach. Orthopedics 2020;43:e369–77. https://doi.org/10.3928/01477447-20200619-03.
